# Dependence of the Sperm/Oocyte Decision on the Nucleosome Remodeling Factor Complex Was Acquired during Recent *Caenorhabditis briggsae* Evolution

**DOI:** 10.1093/molbev/msu198

**Published:** 2014-07-01

**Authors:** Xiangmei Chen, Yongquan Shen, Ronald E. Ellis

**Affiliations:** ^1^Department of Molecular Biology, Rowan University-SOM; ^2^Graduate School of Biomedical Sciences, University of Medicine and Dentistry of New Jersey

**Keywords:** *Caenorhabditis briggsae*, chromatin remodelers, NURF complex, evolution

## Abstract

The major families of chromatin remodelers have been conserved throughout eukaryotic evolution. Because they play broad, pleiotropic roles in gene regulation, it was not known if their functions could change rapidly. Here, we show that major alterations in the use of chromatin remodelers are possible, because the nucleosome remodeling factor (NURF) complex has acquired a unique role in the sperm/oocyte decision of the nematode *Caenorhabditis briggsae*. First, lowering the activity of *C. briggsae* NURF-1 or ISW-1, the core components of the NURF complex, causes germ cells to become oocytes rather than sperm. This observation is based on the analysis of weak alleles and null mutations that were induced with TALENs and on RNA interference. Second, qRT–polymerase chain reaction data show that the *C. briggsae* NURF complex promotes the expression of *Cbr-fog-1* and *Cbr-fog-3*, two genes that control the sperm/oocyte decision. This regulation occurs in the third larval stage and affects the expression of later spermatogenesis genes. Third, double mutants reveal that the NURF complex and the transcription factor TRA-1 act independently on *Cbr-fog-1* and *Cbr-fog-3*. TRA-1 binds both promoters, and computer analyses predict that these binding sites are buried in nucleosomes, so we suggest that the NURF complex alters chromatin structure to allow TRA-1 access to *Cbr-fog-1* and *Cbr-fog-3*. Finally, lowering NURF activity by mutation or RNA interference does not affect this trait in other nematodes, including the sister species *C. nigoni*, so it must have evolved recently. We conclude that altered chromatin remodeling could play an important role in evolutionary change.

## Introduction

To control development, gene expression must be regulated in time and space. The organization of DNA into chromatin plays a key role in this process, by restricting the accessibility of promoters and enhancers (Clapier and Cairns 2009). This restriction also increases precision, because chromatin-remodeling complexes can actively cooperate with transcription factors to control gene expression. However, the pleiotropic phenotypes of most chromatin remodelers have made it difficult to evaluate their roles in evolutionary change.

Nematodes provide an ideal way to address this problem. Most of the known chromatin-remodeling complexes exist in nematodes and control various aspects of development (Cui and Han 2007). Moreover, detailed molecular models have been established for two developmental processes in *Caenorhabditis elegans*. The first involves the specification of sensory rays in the male tail—the trithorax group of chromatin regulators promotes the expression of two Hox genes in the seam cells V5 and V6, whereas the Polycomb group blocks their expression (Chamberlin and Thomas 2000; Ross and Zarkower 2003; Zhang et al. 2003). The other involves the induction of vulval development by an EGF signal from the anchor cell. In the nearby hypodermis, the Nucleosome Remodeling Deacetylase (NURD) and Retinoblastoma (Rb) complexes from the SynMuvB group block the expression of the EGF gene *lin-3* (Cui et al. 2006), preventing the inappropriate activation of the Ras pathway. Other chromatin remodeling complexes help regulate the development of the vulva, but their targets remain unknown (Fay and Yochem 2007). For example, the Tip60/NuA4 histone acetyl transferase (HAT) complex blocks vulval development (Ceol and Horvitz 2004), whereas the nucleosome remodeling factor (NURF) complex promotes vulval cell fates (Andersen et al. 2006).

Chromatin regulators also play an important role in the establishment of the nematode germline (Schaner et al. 2003), and three observations raise the possibility that some of them influence the sperm/oocyte decision. First, the *C. elegans tra-4* gene encodes a PLZF-containing protein that works with histone chaperones and deacetylases to promote many female cell fates (Grote and Conradt 2006), although a role in oogenesis has not been detected. Second, natural variation in the *C. elegans* NATH-10 acetyltransferase controls the number of sperm produced by hermaphrodites (Duveau and Félix 2012), though how it does so remains unknown. Third, the Tip60 HAT complex regulates the sperm/oocyte decision in both *C. elegans* and *C. briggsae* (Guo et al. 2013), although it remains unclear whether Tip60 directly acetylates the transcription factor TRA-1 or works with TRA-1 to acetylate histones in the promoters of targets like *fog-3*. With these cases in mind, we began investigating the role of chromatin remodelers in the sperm/oocyte decision.

This regulatory decision is ideal for comparative evolutionary studies (Haag 2005), because it played a critical role in the origin of self-fertile hermaphrodites (Baldi et al. 2009). Both *C. briggsae* and *C. elegans* evolved hermaphroditic reproduction independently (Cho et al. 2004; Kiontke et al. 2004, 2011). In each species, the *XX* animals have female bodies but make sperm during the L4 larval stage and oocytes as adults, an arrangement that allows self-fertilization. Several studies have shown that this evolutionary step involved independent modifications to the sex-determination pathway, which controls the sperm/oocyte decision. In *C. elegans*, FOG-2 and GLD-1 cause spermatogenesis in hermaphrodites, by blocking the translation of *tra-2* messages (Clifford et al. 2000). By contrast, there is no *fog-*2 gene in *C. briggsae* (Nayak et al. 2005), *gld-1* has a different function (Nayak et al. 2005; Beadell et al. 2011) and *tra-2* is regulated by the novel protein SHE-1 (Guo et al. 2009). The FEM complex is also required for male cell fates in *C. elegans* (Doniach and Hodgkin 1984; Kimble et al. 1984; Hodgkin 1986) but is dispensable for hermaphrodite spermatogenesis in *C. briggsae* (Hill et al. 2006). All of these regulatory genes act through the transcription factor TRA-1 to control *fog-1* and *fog-3*, which promote spermatogenesis in both sexes (Chen and Ellis 2000; Chen et al. 2001; Jin et al. 2001). Because the Tip60 HAT complex is involved in this decision, we began studying the roles of other chromatin remodelers.

Here, we show that NURF-1A and ISW-1, the *C. briggsae* homologs of *Drosophila* NURF301 and ISWI, promote spermatogenesis in both sexes. These proteins are the principal components of the NURF complex (Tsukiyama and Wu 1995; Tsukiyama et al. 1995), which is a member of the imitation switch family of chromatin remodelers (Corona and Tamkun 2004). These complexes use the energy of ATP hydrolysis to slide nucleosomes along the DNA, which increases chromatin fluidity and alters the accessibility of target sites to transcription factors and other regulatory proteins. In *Drosophila*, NURF301 and ISWI are essential for the maintenance of germline stem cells (Cherry and Matunis 2010). In *C. elegans*, they also promote germ cell proliferation and antagonize the Tip60 HAT complex to allow normal vulval development (Andersen et al. 2006). Here, we show that *C. briggsae* NURF-1 and ISW-1 also initiate spermatogenesis, by promoting the expression of *fog-1* and *fog-3*. Furthermore, this regulation is independent of the Gli protein TRA-1. Surprisingly, this role of the NURF complex in the sperm/oocyte decision is unique to *C. briggsae*, although its other functions in embryonic and vulval development have been conserved.

## Results

### The *C. briggsae* NURF Complex Is Required for Germ Cells to Adopt Male Fates

**Fig. 1.**
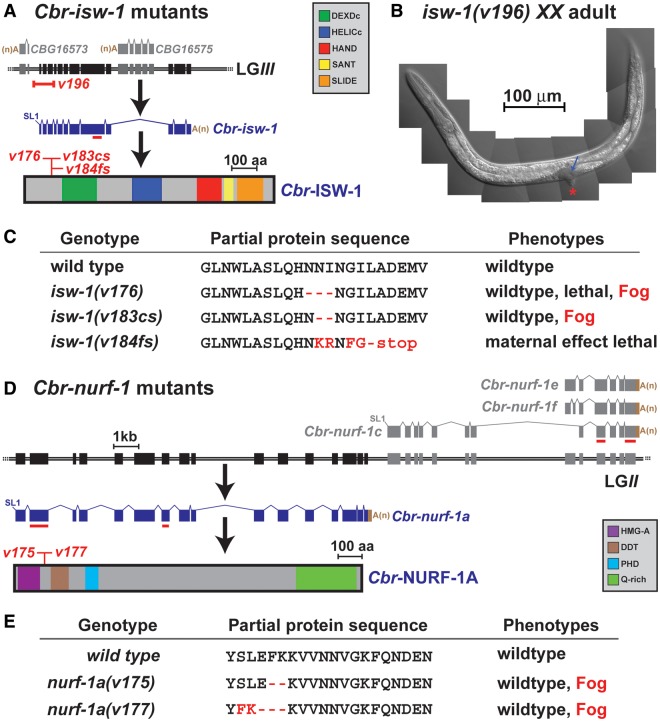
The *Caenorhabditis briggsae* NURF complex regulates the sperm/oocyte decision. (*A*) Location of mutations affecting *Cbr-isw-1. Caenorhabditis briggsae* genomic DNA is shown as a double line, with exons as boxes. Nearby transcripts are above the line in gray, and *isw-1* is below the line, in blue. The position of each new mutation is marked in red, and critical domains of the protein are coded by color. Regions of the transcript targeted by RNAi are underlined in red. (*B*) Nomarski photomicrograph of a *Cbr-isw-1*(*v196*) *XX* adult, showing a protruding vulva (red *) and small germ line (blue arrow). (*C*) Molecular lesions and phenotypes for each *isw-1* mutation (for details, see table 1). (*D*) Location of mutations affecting *Cbr-nurf-1*. Conventions are like those for *isw-1.* (*E*) Molecular lesions and phenotypes for each *nurf-1* mutation (for details, see table 1).

In *Drosophila*, the NURF complex regulates many aspects of development and is critical for the proliferation of germ cells (Alkhatib and Landry 2011). Although the *Drosophila* complex contains four components, ISWI and NURF301 are sufficient to reconstitute its activity (Xiao et al. 2001). In *C. elegans*, mutations in the corresponding NURF genes *isw-1* and *nurf-1* cause sterility and suppress the vulval defects of *lin-15AB* mutants (Andersen et al. 2006). Thus, we focused on orthologs of these genes in the related nematode *C. briggsae*. Using RT-PCR and RACE, we defined the *Cbr-isw-1* and *Cbr-nurf-1* genes (fig. 1*A* and *D*).

To learn the function of the *C. briggsae* NURF complex, we used Transcription Activator-Like Effector Nucleases (TALENs) (Wood et al. 2011; Wei et al. 2014) to make deletions in each gene (fig. 1). We isolated four mutations in *Cbr-isw-1*–*v184*, a frameshifting deletion; *v196*, an 862-bp deletion that removes the first three and a half exons; and *v176* and *v183*, small in-frame deletions. We also recovered two in-frame deletions in *Cbr-nurf-1*–*v175* and *v177.*

Homozygous *isw-1*(*v196*) null mutants had a tiny germ line and a protruding vulva and did not produce offspring (fig. 1*B*). Although *Cbr-isw-1*(*v184*) frameshift mutants were viable if maternal transcripts were present, their progeny died as embryos (fig. 1*C* and table 1). Thus, *C. briggsae* ISW-1 is essential for the development of the vulva and the proliferation of germ cells, and maternal ISW-1 is needed for embryonic viability. Surprisingly, the in-frame mutants of *isw-1* and *nurf-1* were not only healthy but occasionally Fog (Feminization of the germline; fig. 1 and table 1). Thus, the *C. briggsae* NURF complex not only has pleiotropic functions, as in other species, but appears to regulate the sperm/oocyte decision. The Fog phenotype of *C. briggsae* NURF mutants was surprising, because nothing similar had been seen in *C. elegans* (Andersen et al. 2006). Hence, we began a detailed exploration of this trait and then used the results for comparison with other nematode species.

**Table 1.** *Caenorhabditis briggsae* NURF-1 and ISW-1 Promote Spermatogenesis, Viability, and Fertility.

		Germline Phenotypes						
Genotype		Oocytes Only (%)	Sperm and Oocytes (%)	Sperm Only (%)	Immature Germ Cells (%)	Lethal (%)	Other (%)	*n*
*nurf-1*(*v175*)	*XX*	0.8	94.2	0	0	2	3^a^	408
*nurf-1*(*v177*)	*XX*	0.5	93.5	0	0	2	4^a^	336
*isw-1*(*v176*)	*XX*	1	89	0	0	4	6^a^	815
*isw-1*(*v183cs*)	*XX*	11	88	0	0	0	1^b^	67
*isw-1*(*v183cs*)	*XO*	0	0	100	0	0	0	20
*isw-1*(*v184fs*)*^M+^*	*XX*	0	100	0	0	0	0	24
*isw-1*(*v184fs*)*^M^^−^*	*XX*	0	0	0	0	100	0	52
*isw-1*(*v184fs*)/+	*XO*^c^	0	0	99^d^	0	0	1^e^	101
*isw-1*(*v196df*)	*XX*	0	0	0	100^f^	0	0	16
*isw-1*(*v196df*)/+	*XO*^g^	0	4	96	0	0	0	80
*isw-1*(*v196df*)	*XO*	0	0	0	100^h^	0	0	21

Note.—Animals were scored at 20 °C, except *isw-1*(*v183*cs), which was scored at the restrictive temperature of 15 °C.

^a^Egg-laying defective.

^b^Disorganized germ line.

^c^Males were progeny from crosses of *dpy-18* hermaphrodites with *v184/dpy-18* or *v184* males.

^d^One male had sperm at both ends of the gonad. Five males made very few sperm.

^e^The male had a ruptured gonad, produced sperm, and appeared to be starting oogenesis.

^f^All animals were smaller in soma and germ line than AF16, and had a protruding vulva.

^g^Males were progeny from crosses of *dpy-18* hermaphrodites with *v196/dpy-18* or *v196* males.

^h^All animals were smaller in soma and germ line, and had a blunt, defective tail.

### The *C. briggsae* NURF Complex Is Required to Initiate Spermatogenesis in Both Sexes

**Fig. 2.**
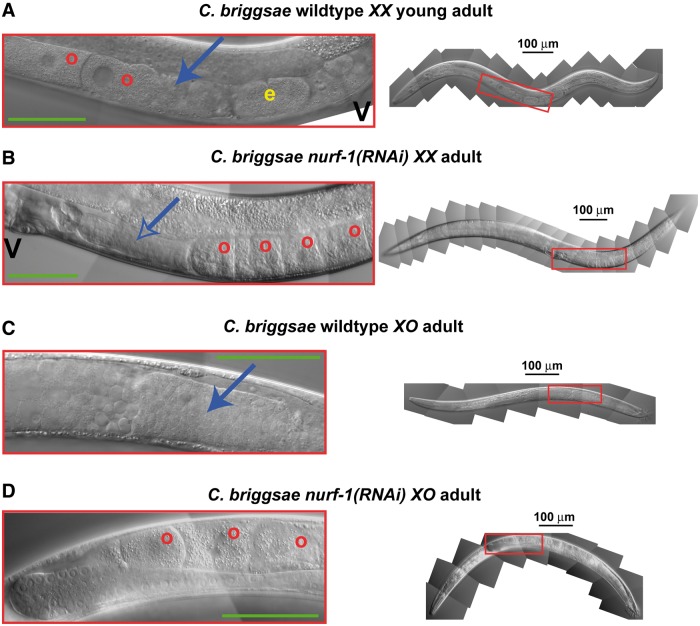
*Caenorhabditis briggsae* NURF-1 controls the sperm/oocyte decision in both sexes. (*A*) *Caenorhabditis briggsae* wild-type *XX* young adult. (*B*) *Caenorhabditis briggsae nurf-1*(*RNAi*) *XX* adult. This animal is older than the control in panel A, because the *nurf-1* germ line develops more slowly. (*C*) *Caenorhabditis briggsae* wild-type male. (*D*) *Caenorhabditis briggsae nurf-1*(*RNAi*) Fog male. In all panels, anterior is left and ventral is down. The size of each inset is shown by a box on the adjacent animal; the green bar in the inset is 50 µm. Finally, “o” indicates oocytes, “e” embryos, “V” the vulva, a solid blue arrow marks a spermatheca filled with sperm, and a hollow blue arrow marks an empty spermatheca.

Because weak alleles of *nurf-1* and *isw-1* caused a Fog phenotype in some animals, we used RNA interference to lower gene activity further, without eliminating it altogether (Montgomery et al. 1998). Knocking down either *Cbr-nurf-1* or *Cbr-isw-1* caused animals of both sexes to make oocytes instead of sperm (fig. 2 and supplementary fig. S1, Supplementary Material online). This effect was complete in *XX* animals and highly penetrant in *XO* males (tables 2 and 3). As in *C. elegans*, RNAi against the remaining NURF components was lethal (tables 2 and 3). Thus, the *C. briggsae* NURF complex is required in both sexes for germ cells to initiate spermatogenesis rather than oogenesis.

**Table 2.** *Caenorhabditis briggsae* NURF-1A Controls Germ Cell Fates in Both Sexes.

Targets of RNAi		Oocytes Only (%)	Sperm and Oocytes (%)	Sperm Only (%)	Other (%)	*n*
*Cbr-nurf-1a*	*XX*	99.6	0.4	0	0	421
*Cbr-nurf-1a*	*XO*	62	9	20	9^a^	44
*Cbr-nurf-1cef*^b^	*XX*	0	100	0	0	207
*Cbr-nurf-1a* + *nurf-1cef*	*XX*	86	12	0	2^c^	253

^a^Undifferentiated germ cells, some with vacuoles in the germ line.

^b^RNAi was used to simultaneously target the *nurf-1.2c*, *e*, and *f* transcripts.

^c^Dead eggs.

**Table 3.** *Caenorhabditis briggsae* ISW-1 Acts with NURF-1A to Control Germ Cell Fates.

*Drosophila* Homolog	Target of RNAi		Oocytes Only (%)	Sperm and Oocytes (%)	Sperm Only (%)	Dead Eggs (%)	Dead Larvae (%)	*n*
ISWI	*Cbr-isw-1*	*XX*	100	0	0	0	0	398
ISWI	*Cbr-isw-1*	*XO*	52	18	30	NA	NA	33
NURF55	*Cbr-lin-53*	*XX*	0	1^a^	0	91	8	274
NURF55	*Cbr-rba-1*	*XX*	0	3	0	96	1	125
NURF38	*Cbr-pyp-1*	*XX*	0	0	0	23	77	150

^a^Two animals produced both sperm and oocytes but were sterile.

To see whether the oocytes made by these mutants were functional, we crossed individual Fog animals with wild-type males and studied the eggs they laid over the following 20 h. We found that 92% of the progeny from *Cbr-nurf-1*(*RNAi*) mothers hatched and grew into healthy adults, and only 8% died as embryos (*n* = 751). Similarly, 86% of the progeny from *Cbr-isw-1*(*RNAi*) mothers became healthy adults, and 14% died as embryos (*n* = 280). As a control, we found that 7% of the progeny from *she-1*(*v35*) mothers died as embryos (*n* = 242). This level of lethality is similar to that observed for the first oocytes fertilized in *C. elegans* Fog mothers (Andux and Ellis 2008). Thus, the oocytes of *Cbr-nurf-1*(*RNAi*) and *Cbr-isw-1*(*RNAi*) Fog animals function normally.

### A Single NURF-1 Product Regulates the Sperm/Oocyte Decision in *C. briggsae*

Alternative splicing produces several NURF301 isoforms in *Drosophila* (Kwon et al. 2009). When we used RT-PCR to characterize the *C. briggsae* messages, we detected four *nurf-1* transcripts (fig. 2*D*), which correspond to prominent *nurf-1* transcripts identified in *C. elegans* (Andersen et al. 2006). One message is produced from the left half of the *nurf-1* locus, which we name *nurf-1A*. The remaining transcripts are produced from the right half of the locus, which we call *nurf-1B*. Neither *C. briggsae* nor *C. elegans* makes a product like full-length *Drosophila* NURF301, which would span the entire region (Xiao et al. 2001; Andersen et al. 2006), so the range of NURF-1 isoforms differs from that seen in fruit flies.

Both *C. elegans* and *C. briggsae* share *nurf-1a*, *c*, and *e* transcripts (Andersen et al. 2006)*.* Although knocking down *nurf-1A* caused a Fog phenotype, knocking down the remaining transcripts did not (tables 2 and 3). Furthermore, we could detect no other defects in the *nurf-1B*(*RNAi*) animals, and mutations that affect the corresponding *C. elegans* transcripts also had no phenotype (Andersen et al. 2006). Thus, *nurf-1a* might encode the only NURF-1 isoform that regulates developmental decisions in nematodes. NURF-1A is also the only isoform to contain an HMG-A domain, which is known to bind DNA (Travers 2000).

As in *C. elegans*, *Cbr-isw-1* is trans-spliced to SL1 and produces a single transcript. When we used RT-PCR to study *nurf-1a*, *isw-1*, and *lin-53* transcript levels, we found that each was predominantly expressed in germ cells, as expected from the phenotypes we observed (supplementary fig. S2, Supplementary Material online).

### NURF-1A and ISW-1 Promote the Expression of the Male Genes *fog-1* and *fog-3*

In *C. elegans*, *fog-1* and *fog-3* are required for germ cells to become sperm rather than oocytes (Barton and Kimble 1990; Ellis and Kimble 1995), and the function of each gene is conserved in *C. briggsae* (Chen et al. 2001). Because the NURF complex is predicted to regulate transcription by altering chromatin (Tsukiyama and Wu 1995), we used real-time RT-PCR to study these transcripts in wild-type, *Cbr-nurf-1a*(*RNAi*) and *Cbr-isw-1*(*RNAi*) animals.

**Fig. 3.**
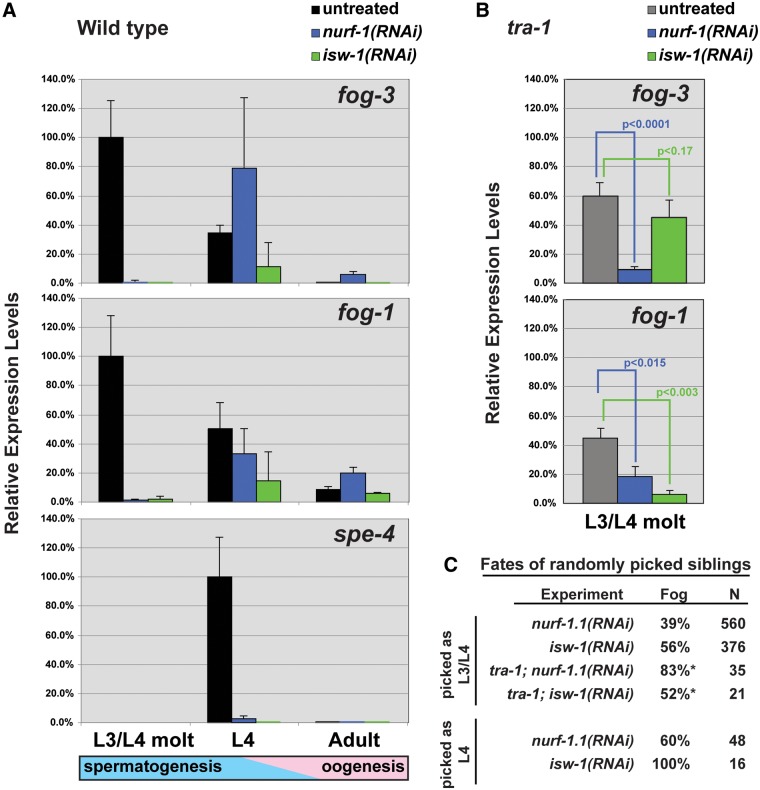
*Caenorhabditis briggsae* NURF-1A and ISW-1 control the expression of *fog-1* and *fog-3*. Transcript levels were measured by real-time RT-PCR and calculated using 2exp(−ΔΔ*C*_T_), with *rpb-1* transcripts as a reference; *fog-1* and *fog-3* were normalized to wild-type animals at the *XX* L3/L4 molt and *spe-4* to the *XX* L4. Error bars represent standard error of the mean. (*A*) Wild-type animals subjected to RNAi. (*B*) *tra-1*(*nm2*) animals subjected to RNAi. *P* values were determined with a Student’s *t*-test, one-tailed, with unequal variance. (*C*) The phenotypes of siblings of animals that were collected for transcript analysis.

In *C. elegans* hermaphrodites, *fog-1* and *fog-3* expression is high during larval development, when germ cells are undergoing spermatogenesis, and low in adults, during oogenesis (Chen and Ellis 2000; Lamont and Kimble 2007). Thus, we focused on three developmental stages of *C. briggsae XX* animals: larvae at the L3/L4 molt, L4 larvae, and adults. The levels of *Cbr-fog-1* and *Cbr-fog-3* transcripts were highest at the L3/L4 molt (fig. 3*A*), when germ cells were beginning to select male fates (Lee et al. 2011; Morgan et al. 2013). In L4 larvae, the levels of *Cbr-fog-1* and *Cbr-fog-3* transcripts had begun to decline, but genes like *Cbr-spe-4*, which are active in primary spermatocytes (Arduengo et al. 1998; Gosney et al. 2008), were expressed at high levels. In adults, *Cbr-fog-1*, *Cbr-fog-3*, and *Cbr-spe-4* transcripts were all found at low or undetectable levels.

However, in both *Cbr-nurf-1a*(*RNAi*) and *Cbr-isw-1*(*RNAi*) animals, the levels of *fog-1* and *fog-3* transcripts were dramatically lower at the L3/L4 molt, a period that is critical for the initiation of spermatogenesis (fig. 3*A*). By the L4 stage, the levels of *fog-1* and *fog-3* transcripts were nearly wild type, but this recovery was too late to alter development, since about half of the animals only produced oocytes (fig. 3*C*). This Fog phenotype was confirmed by the absence of *spe-4* transcripts in the *Cbr-nurf-1a*(*RNAi*) and *Cbr-isw-1*(*RNAi*) L4 larvae (fig. 3*A*). Finally, *fog-1* and *fog-3* transcripts were found at higher levels in *Cbr-nurf-1a*(*RNAi*) adults than in the wild type (*P* < 0.003, fig. 3*A*). Thus, NURF-1A and ISW-1 promote high levels of *fog-1* and *fog-3* expression in larvae, but NURF-1A might help repress these transcripts in adults.

### The NURF Complex and TRA-1 Act Independently on *fog-1* and *fog-3*

TRA-1 is a Gli transcription factor that acts at the end of the sex-determination pathway (Hodgkin and Brenner 1977; Zarkower and Hodgkin 1992; Kelleher et al. 2008). Furthermore, there are conserved TRA-1 binding sites in the *fog-1* and *fog-3* promoters, and *C. elegans* TRA-1 can bind these sites in vitro (Chen and Ellis 2000; Jin et al. 2001). To see if the NURF complex acts upstream of TRA-1 to promote the expression of *fog-1* and *fog-3*, we examined double mutants with *Cbr-tra-1*(*nm2*), a nonsense mutation (Kelleher et al. 2008), and *Cbr-tra-1*(*v181*), which causes a frameshift prior to the zinc finger domain (see Materials and Methods). These null mutations transform *XX* animals into males that make sperm as larvae and oocytes as adults. Surprisingly, *Cbr-tra-1*; *Cbr-nurf-1a*(*RNAi*) and *Cbr-tra-1*; *Cbr-isw-1*(*RNAi*) *XX* young adults only made oocytes (table 4). Thus, NURF-1A and ISW-1 can promote spermatogenesis even in the absence of TRA-1 and must act near the end of the sex-determination pathway in germ cells.

**Table 4.** NURF-1A and ISW-1 Are Epistatic to TRA-1.

Genetic Background	Target Gene	Oocytes Only (%)	Sperm and Oocytes (%)	Sperm Only (%)	*n*
*tra-1*(*nm2*)^a^		0	96	4	25
*tra-1*(*nm2*)^a^	*nurf-1a*	86	8	4	48
*tra-1*(*nm2*)^a^	*isw-1*	56	44	0	50
*tra-1*(*v181*fs)^a^		0	88	12	60
*tra-1*(*v181*fs)^a^	*nurf-1a*	100	0	0	122
*tra-1*(*v181*fs)^a^	*isw-1*^b^	87	13	0	160

^a^*XX tra-1* animals were self-progeny of *tra-1 +/ +dpy-18* mothers. They were picked as L4s and scored with DIC 1 day later.

^b^Four micrograms per microliter *isw-1* dsRNA was injected.

We also studied double mutants with *Cbr-tra-2*. The weak allele *tra-2*(*nm9*ts) and NURF complex RNAi mutually suppressed each other (table 5), which is consistent with models in which TRA-1 and the NURF complex act independently on the *fog* promoters. NURF RNAi also caused oogenesis in *tra-2*(*nm1*) null mutants, which normally make only sperm. Over 50% of the *Cbr-tra-2*(*nm1*); *Cbr-nurf-1a*(*RNAi*) animals and some *Cbr-tra-2*(*nm1*); *Cbr-isw-1*(*RNAi*) animals made oocytes, but we never saw oogenesis in the controls (table 5).

**Table 5.** Mutations in the NURF Complex and *Cbr-tra-2* Are Mutually Antagonistic.

Genetic Background	Target Gene	Fog (%)	Sperm Only (%)	Other^a^ (%)	*n*
*tra-2*(*nm9*ts)		0	100	0	21
*tra-2*(*nm9*ts)	*nurf-1a*	55	45	0	22
*tra-2*(*nm9*ts)	*isw-1*^b^	32	42	26	31
*tra-2*(*nm1*) *cby-15*^c^		0	100	0	36
*tra-2*(*nm1*) *cby-15*	*nurf-1a*	50	38	12	24
*tra-2*(*nm1*) *cby-15*	*isw-1*	0	100	0	195
*tra-2*(*nm1*)		0	96	4	25
*tra-2*(*nm1*)	*nurf-1a*	62	32	6	72
*tra-2*(*nm1*)	*isw-1*	3	55	42	77
*tra-2*(*nm1*)	*isw-1*^b^	3	76	21	38

^a^Animals had defective germ lines with no differentiated cells. Some had vacuoles.

^b^Four micrograms per microliter *isw-1* dsRNA was injected.

^c^cby-15 was used as a linked marker for tra-2 in this set of crosses.

To confirm these results, we studied *Cbr-tra-1*(*nm2*); *nurf-1a*(*RNAi*) and *Cbr-tra-1*(*nm2*); *isw-1*(*RNAi*) mutants at the L3/L4 molt. Both strains had lower levels of *fog-1* and *fog-3* transcripts than *tra-1* controls (fig. 3*B*). Because NURF-1A and ISW-1 do not require TRA-1 to regulate these genes, the NURF complex is likely to regulate their promoters independently.

### The Role of the NURF Complex Changed during Recent Nematode Evolution

In *C. elegans*, mutations that affect the NURF complex did not block spermatogenesis (Andersen et al. 2006), whereas in *C. briggsae,* lowering NURF activity even slightly feminized the germ line. To see whether the *C. elegans* NURF complex plays any role in the sperm/oocyte decision, we performed more detailed studies of one *isw-1* mutant*.* The *Cel-isw-1*(*n3297*) mutation alters an amino acid located just after the DEXDc domain (Andersen et al. 2006), whereas the *Cbr-isw-1*(*v176*) and *v183* mutations delete a few amino acids just before this domain. All of these mutants are viable and none show the complete sterility of null alleles, so they each cause a partial loss of function. We found that 38% of *Cel-isw-1*(*n3297*) *XX* mutants were sterile, but none of them appeared Fog (*n* = 213). Furthermore, we examined some of the sterile animals with differential interference contrast (DIC) optics, and 67% had sperm and defective oocytes, whereas the rest had small germ lines without differentiated gametes (*n* = 33). Finally, 90% of *Cel-isw-1*(*n3297*) *XO* males made sperm, 4% had tumorous germ lines, and 3% had small germ lines but none made oocytes (*n* = 49). Since these *C. briggsae* and *C. elegans* mutations all decrease ISW-1 activity, it seems likely that lowering the activity of the NURF complex alters the sperm/oocyte decision in *C. briggsae* but not in *C. elegans*.

Second, we compared null alleles. The *Cbr-isw-1*(*v196*) deletion is a null allele (supplementary fig. S3, Supplementary Material online). Although homozygotes are sterile because of severe germ line defects, some *Cbr-isw-1*(*v196*)/+ males made oocytes (supplementary fig. S4, Supplementary Material online, and table 1). By contrast, no *Cel-isw-1*(*ok1951*)/+ males made oocytes (*n* = 131). This difference is significant at *P* = 1%, in a *z*-test comparing the two proportions.

**Fig. 4.**
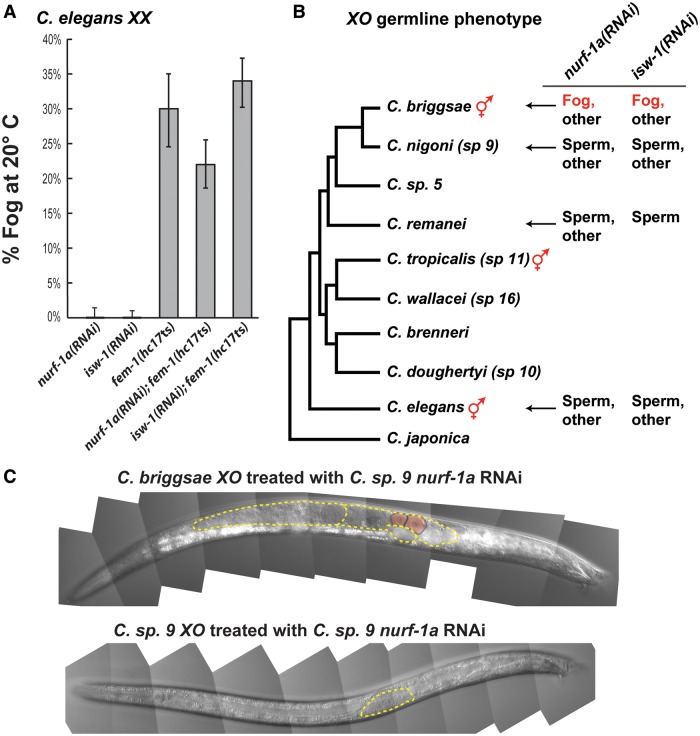
The role of NURF-1 and ISW-1 in germ cells changed during evolution. (*A*) At 20 °C, *Caenorhabditis elegans* NURF-1A or ISW-1 were knocked down by RNAi in *fem-1*(*hc17*ts) mutants. The double mutants were not more feminized than *fem-1*. Error bars represent 95% confidence intervals for a proportion. (*B*) Phylogeny of *Caenorahbditis* (Kiontke et al. 2011; Felix et al. 2014) showing the results of RNAi experiments (supplementary table S2, Supplementary Material online). (*C*) *Caenorhabditis nigoni* dsRNA causes a Fog phenotype in *C. briggsae* males (top) but not in *C. nigoni* (bottom). Gonads are outlined with dotted yellow lines, and two oocytes are shaded pink.

Third, we studied the function of the NURF complex in mutants that were predisposed to producing oocytes. Specifically, we used RNAi to knock down *Cel-nurf-1a* or *Cel-isw-1* in either *Cel-fog-1*(*q253*ts) or *Cel-fem-1*(*hc17*ts) mutants, at the intermediate temperature of 20 °C. Although NURF RNAi did not cause synthetic feminization (fig. 4 and supplementary table S1, Supplementary Material online), similar experiments using *trr-1* caused strong synthetic feminization (Guo et al. 2013). Thus, none of these studies identified any role for the NURF complex in the *C. elegans* sperm/oocyte decision.

Finally, we studied the NURF complex in male/female species related to *C. briggsae*. Knocking down either *nurf-1a* or *isw-1* in *C. nigoni* or *C. remanei* did not cause males to produce oocytes but did cause occasional sterility (fig. 4 and supplementary table S2, Supplementary Material online)*.* In a control experiment, *fog-3*(*RNAi*) caused 42% of *C. nigoni* males to become Fog (*n* = 38), so this cell-fate decision is sensitive to RNA interference. Finally, when we injected *C. nigoni nurf-1a* or *isw-1* dsRNA into *C. briggsae* wild-type strains, each one caused a Fog phenotype in males (fig. 4*C* and supplementary table S2, Supplementary Material online). Thus, the NURF complex has either acquired a new function in *C. briggsae*, or its role in the sperm/oocyte decision has changed so much that it can only be detected in *C. briggsae*.

## Discussion

### *Caenorhabditis briggsae* NURF-1A and ISW-1 Are Required for Germ Cells to Adopt Male Fates

In *C. briggsae*, knocking down either NURF-1A or ISW-1 by RNA interference causes animals of both sexes to make oocytes instead of sperm. Furthermore, partial loss-of-function mutations in either gene cause some animals to become Fog, and loss of just one copy of *isw-1* causes rare males to make oocytes. Thus, NURF-1A and ISW-1 promote spermatogenesis in *C. briggsae*, and small decreases in their activities cause germ cells to differentiate as oocytes. These two proteins are likely to work together as part of a NURF chromatin remodeling complex.

### The Role of the NURF Complex in Germ Cells Changed during Recent Evolution

This role of the NURF complex in the sperm/oocyte decision appears to be unique to *C. briggsae*. In *C. elegans*, *nurf-1* and *isw-1* mutants make both sperm and oocytes (Andersen et al. 2006). Furthermore, lowering *C. elegans* NURF activity does not change the sperm/oocyte decision in mutants that are predisposed to a transformation in germ cell fates, whereas lowering Tip60 HAT activity causes synthetic feminization in these genetic backgrounds (Guo et al. 2013). Finally, *C. elegans isw-1*(*null*)/+ males do not make oocytes, unlike some of their *C. briggsae* counterparts.

We also found no role for the NURF complex in the sperm oocyte decision of male/female nematodes. *Caenorhabditis nigoni* is so closely related to *C. briggsae* that they can mate and produce viable offspring (Woodruff et al. 2010), and these species appear to have diverged on the order of 10^7^ generations ago (Cutter et al. 2010). However, knocking down NURF activity in *C. nigoni* did not cause males to make oocytes, and similar experiments with the more distant relative *C. remanei* had the same result. We know the dsRNA we used was of high quality, because *C. briggsae* germ cells became oocytes in response to both *C. nigoni nurf-1a* and *C. nigoni isw-1* dsRNA, even though *C. nigoni* germ cells did not. Furthermore, these differences were not due to variable sensitivity to RNA interference, because knocking down NURF activity in *C. nigoni* and *C. remanei* strongly affected germline traits like proliferation. We conclude that the role of the NURF complex changed during recent evolution.

**Fig. 5.**
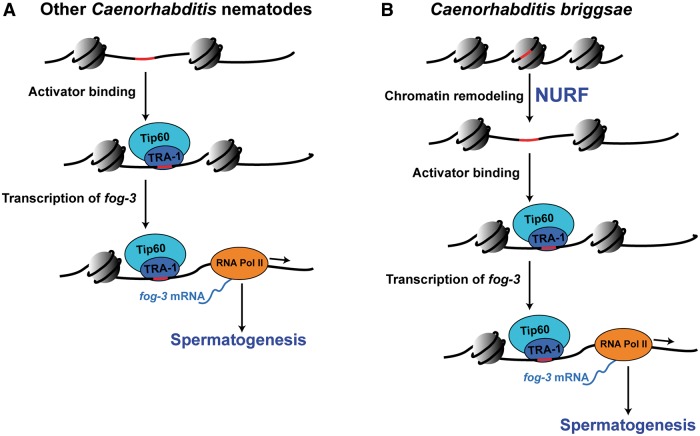
Model for the initiation of spermatogenesis in *Caenorhabditis.* (*A*) In most species, the NURF complex is not required. For spermatogenesis, an unknown activator promotes the expression of *fog-3.* Currently, the only candidate for this activator is full-length TRA-1, perhaps in association with the Tip60 HAT complex (Guo et al. 2013). (*B*) In *C. briggsae*, the NURF complex is required for *fog-3* to promote spermatogenesis. Because NURF is known to remodel chromatin, the simplest possibility is that it opens up the *fog-3* promoter, so that TRA-1 binding sites (shown in red) are accessible. TRA-1 then works with the Tip60 HAT complex to promote expression of *fog-3*, which works with *fog-1* to cause germ cells to initiate spermatogenesis.

Two types of models could explain this change. In the first, the *C. briggsae* NURF complex was recruited to regulate the sperm/oocyte decision; NURF-1A might be critical for this function because it has an HMG-A domain, which can bind DNA. In the most parsimonious version, the configuration of chromatin in this region of the genome has be opened in *C. briggsae* larvae but is already accessible in *C. elegans* larvae (fig. 5)*.* In the figure, we propose that the NURF complex allows TRA-1 access to the *fog* promoters, because it is the only transcription factor known to interact with them; however, it remains possible that an unknown activator regulates these genes. This model is consistent with data from Berkseth et al. (2013), who showed that TRA-1 binds the *fog-3* promoter at higher levels in *C. elegans* than in *C. briggsae*. However, the underlying differences responsible for altered chromatin structure remain unknown, because the *C. briggsae* and *C. nigoni fog-3* promoters are similar in sequence, and the *C. briggsae fog-3* promoter is regulated normally in *C. elegans* (Chen et al. 2001)*.*

In the second type of model, *C. briggsae* changed, so that it now depends on NURF activity, which is not needed for spermatogenesis in other worms. For example, *C. briggsae* might have lost a redundant chromatin regulator that controls the *fog-3* locus, which made the NURF complex essential in that species. In either scenario, broad changes in the regulation of chromatin structure have occurred during short evolutionary time spans, and these changes played important roles in shaping the expression of genes such as *fog-1* and *fog-3*.

Although chromatin states can be assayed directly in single cells like yeast (Boeger et al. 2008; Yen et al. 2012), such experiments are more difficult in multicellular organisms. In this case, only 75 germ cells in young L4 hermaphrodites will become spermatocytes, out of a total of about 1,500, and these cells cannot be isolated from the rest of the animal. Thus, distinguishing among these models will require a new array of tools. We expect genome editing might help create these tools (Wood et al. 2011; Lo et al. 2013; Wei et al. 2014) and will become a standard part of evolutionary studies in the future.

### NURF-1A Is a Critical Component of the Nematode NURF Complex

In *Drosophila*, NURF301 and ISWI are core components of the NURF complex, which also contains NURF38 and NURF55. In *C. briggsae*, mutants in NURF-1A and ISW-1 have similar phenotypes, so they probably form a nematode NURF complex. Because the homologs of NURF38 and NURF55 are essential, they could not be tested for a role in the sperm/oocyte decision but probably also form part of this complex. Because null mutations in *isw-1* block germ cell proliferation, at least one function of the NURF complex is broadly conserved in animals.

However, the structure of the NURF complex remains unclear. In *Drosophila*, *nurf301* produces multiple transcripts, whose differing roles are poorly understood (Kwon et al. 2009). In *C. elegans*, only NURF-1A regulates vulval cell fates (Andersen et al. 2006), and in *C. briggsae*, only NURF-1A regulates germ cell fates. Inactivating other *nurf-1* products causes no phenotype either species. Because Cbr-NURF-1A, Cel-NURF-1A, and *Drosophila* NURF301C are similar in structure to each other and to the human protein FAC1 (Bowser et al. 1995), they might share a conserved chromatin regulatory function.

### *Caenorhabditis briggsae fog-3* Is Ideal for Studying Interactions of Gli Proteins with Chromatin Regulators

Developing germ cells make two major decisions: Whether to proliferate or enter meiosis, and whether to become spermatocytes or oocytes (Kimble and Crittenden 2007; Morgan et al. 2013). In nematodes, FOG-1 and FOG-3 influence the mitosis–meiosis decision and are absolutely required for germ cells to adopt male fates (Barton and Kimble 1990; Ellis and Kimble 1995; Thompson et al. 2005; Snow et al. 2013). Three sets of proteins are known to regulate the expression of these genes. First, TRA-1 is the sole nematode Gli protein. Cleaved TRA-1 favors oogenesis by repressing *fog-1* and *fog-3*, whereas full-length TRA-1 appears to favor spermatogenesis by promoting their expression (Chen and Ellis 2000; Schvarzstein and Spence 2006). Second, the Tip60 HAT complex is required for TRA-1 to promote *fog-3* expression (Guo et al. 2013). It might act on TRA-1 or work with TRA-1 to modify chromatin. Third, NURF activity is needed in *C. briggsae* larvae for the expression of *fog-1* and *fog-3*, when germ cells are beginning to commit to spermatogenesis.

Although TRA-1 acts directly on the *fog-3* promoter, knocking down NURF activity lowers the expression of *fog-1* and *fog-3* in *tra-1*(*null*) mutants. Thus, the NURF complex must act downstream of TRA-1, and we infer that TRA-1 and the NURF complex act independently on these two promoters. This idea is supported by one other observation—*Cbr-tra-2*(*null*) mutants partially suppress the *Cbr-nurf-1a*(*RNAi*) and *Cbr-isw-1*(*RNAi*) phenotypes. Because these *tra-2* mutations act through TRA-1 to promote spermatogenesis, they might increase its activator function enough to promote *fog-3* expression without chromatin remodeling. Thus, we favor models in which the NURF complex remodels chromatin to provide TRA-1 and Tip60 access to the *fog-3* promoter (fig. 5). This possibility is consistent with predictions that the TRA-1 binding sites in the *Cbr-fog-1* and *Cbr-fog-3* promoters are normally buried in nucleosomes (supplementary fig. S5, Supplementary Material online; Xi et al. 2010) and might require NURF activity to become accessible.

In yeast and humans, HAT complexes and chromatin remodelers can work as coactivators to regulate gene expression (Featherstone 2002). Furthermore, studies in *Drosophila* showed that the Gcn5 HAT complex and the NURF complex regulate a common set of target genes, and that the NURF complex is required for Gcn5 to access these targets (Carre et al. 2008). Because the Gli protein TRA-1, the NURF chromatin-remodeling complex, and Tip60 HAT complex all regulate *fog-3* expression in *C. briggsae*, this system provides an ideal model for exploring how chromatin-remodelers interact with Gli transcription factors to control target genes.

## Materials and Methods

### Strains and Genetics

*Caenorhabditis briggsae* mutants were derived from the wild isolate AF16 (Fodor et al. 1983). They include LG*II*: *dpy*(*nm4*) (Hill et al. 2006), *tra-2*(*nm1*) and *tra-2*(*nm9*ts) (Kelleher et al. 2008), and *cby-15*(*sy5418*) (Sternberg P, personal communication); LG*III*: *tra-1*(*nm2*) (Kelleher et al. 2008) and *dpy-18*(*mf104*) (Felix M, personal communication); and LG*IV: she-1*(*v49*) (Guo et al. 2009). We also used the *C. briggsae* wild isolate HK104 (Kagawa H, personal communication; Stein et al. 2003).

*Caenorhabditis elegans* mutants were derived from the wild isolate N2 (Brenner 1974) and include LG*I: fog-1*(*q253*ts) (Barton and Kimble 1990), LG*III: isw-1*(*ok1951*) (Andersen et al. 2006), and LG*IV: fem-1*(*hc17*ts) (Nelson et al. 1978)*.* All *C. nigoni* RNAi experiments were done using JU1421 (Felix M, personal communication), and *C. remanei* RNAi experiments were done using PB4641 (Baird S, personal communication).

### RNA Interference

Each template was amplified from mixed-stage cDNA by the PCR, using primers that contained a T7 promoter (supplementary table S3, Supplementary Material online). Templates were purified with a PCR Purification kit (Qiagen) and transcribed using MegaScript (Ambion). After annealing, double-stranded RNA was purified with MegaClear (Ambion). RNAi was performed by injection, using 1 µg/µl solutions of dsRNA (Fire et al. 1998).

### Semiquantitative RT-PCR

For each genotype, groups of five adult worms of the desired age and phenotype were collected and processed as described (Chen and Ellis 2000); three independent samples were prepared to confirm reproducibility. RT-PCR was performed as described, using HotMaster Taq DNA polymerase (5PRIME) and MMLV Reverse Transcriptase (Invitrogen). The PCR reactions were run for 35 cycles using primers from supplementary table S4*A*, Supplementary Material online.

### Real-Time Quantitative RT-PCR

Groups of five worms of the desired age and phenotype were picked and prepared as described above, and three or four independent biological replicates and two technical replicates were assayed for each data point. Siblings of the collected animals were allowed to mature and scored for phenotype, to determine the efficiency of the RNAi. For each reaction, 1/20 of the total cDNA sample was used in a final volume of 25 µl, which included 12.5 µl of FastStart Universal SYBR Green Master (Rox) (Roche) and 6 µM primers (supplementary table S4*B*, Supplementary Material online). Amplification was 40 cycles, using Applied Biosystems 7500 Real-Time PCR Systems. Samples that did not show detectable amplification by the final cycle were arbitrarily assigned a *C*_t_ value of 40.

### Microscopy

Worms were observed with DIC microscopy. Images were captured with a Zeiss Axiocam digital camera and Zeiss AxioVision software, and assembled using Adobe Photoshop.

### Determination of Gene Structures

For *isw-1*, *nurf-1a*, and *nurf-1c*, *e,* and *f*, 3′-RACE, 5′-RACE (Frohman 1994), and RT-PCR with an SL1 primer were used to identify the ends of each transcript. The internal splice sites were identified by RT-PCR. Because of the duplication and specialization of two exons, the genomic sequences that encode the *C. briggsae nurf-1a* transcript do not overlap those that encode the remaining *nurf-1* transcripts. We are naming this entire complex locus “*nurf-1*” to be consistent with the literature from other species. The portion that encodes the *nurf-1a* transcript is officially the *nurf-1A* gene. The portion that encodes the remaining *nurf-1* transcripts is *nurf-1B*.

### TALEN Knockout Mutants

TALENs were designed and produced as described by Wei et al. (2014). To create mutants, mRNA was injected into the gonad of adult hermaphrodites (Wood et al. 2011). The injection solution contained a pair of TALEN mRNAs, each at 3 µg/µl. At 20 °C, the F_1_ progeny from a 6- to 32-h time window were singled to new plates at the L4 stage, and F_2_ animals that were heterozygous or homozygous for new mutations were identified by phenotype or by PCR analysis of the target site.

The *v181* mutation deletes nucleotides 276–280 from the coding region of *tra-1a*, creating a frameshift and early stop codon. As a consequence, it also removes nt 150–154 of *tra-1b.* Because *v181* eliminates the products of both *tra-1* transcripts, it must be a null allele.

### Fertility and Oocyte Quality Assays

To assess fertility and oocyte quality for *nurf-1a*(*RNAi*) and *isw-1*(*RNAi*) Fog mutants, ten individuals were singled and each crossed with six wild-type males for 24 h at 20 °C. Afterward, they were scored for fertility, and their progeny for lethality.

### Statistical Analyses

To determine the significance of the difference between two proportions, we used www.vassarstats.net/propdiff_ind.html (last accessed June 30, 2014) to calculate the *z*-ratio and *P* values.

## Supplementary Material

Supplementary figures S1–S5 and tables S1–S4 are available at *Molecular Biology and Evolution* online (http://www.mbe.oxfordjournals.org/).

Supplementary Data

## References

[msu198-B1] Alkhatib SG, Landry JW (2011). The nucleosome remodeling factor. FEBS Lett..

[msu198-B2] Andersen EC, Lu X, Horvitz HR (2006). *C. elegans* ISWI and NURF301 antagonize an Rb-like pathway in the determination of multiple cell fates. Development.

[msu198-B3] Andux S, Ellis RE (2008). Apoptosis maintains oocyte quality in aging *Caenorhabditis elegans* females. PLoS Genet..

[msu198-B4] Arduengo PM, Appleberry OK, Chuang P, L’Hernault SW (1998). The presenilin protein family member SPE-4 localizes to an ER/Golgi derived organelle and is required for proper cytoplasmic partitioning during *Caenorhabditis elegans* spermatogenesis. J Cell Sci..

[msu198-B5] Baldi C, Cho S, Ellis RE (2009). Mutations in two independent pathways are sufficient to create hermaphroditic nematodes. Science.

[msu198-B6] Barton MK, Kimble J (1990). *fog-1*, a regulatory gene required for specification of spermatogenesis in the germ line of *Caenorhabditis elegans*. Genetics.

[msu198-B7] Beadell AV, Liu Q, Johnson DM, Haag ES (2011). Independent recruitments of a translational regulator in the evolution of self-fertile nematodes. Proc Natl Acad Sci U S A..

[msu198-B8] Berkseth M, Ikegami K, Arur S, Lieb JD, Zarkower D (2013). TRA-1 ChIP-seq reveals regulators of sexual differentiation and multilevel feedback in nematode sex determination. Proc Natl Acad Sci U S A..

[msu198-B9] Boeger H, Griesenbeck J, Kornberg RD (2008). Nucleosome retention and the stochastic nature of promoter chromatin remodeling for transcription. Cell.

[msu198-B10] Bowser R, Giambrone A, Davies P (1995). FAC1, a novel gene identified with the monoclonal antibody Alz50, is developmentally regulated in human brain. Dev Neurosci..

[msu198-B11] Brenner S (1974). The genetics of *Caenorhabditis elegans*. Genetics.

[msu198-B12] Carre C, Ciurciu A, Komonyi O, Jacquier C, Fagegaltier D, Pidoux J, Tricoire H, Tora L, Boros IM, Antoniewski C (2008). The *Drosophila* NURF remodelling and the ATAC histone acetylase complexes functionally interact and are required for global chromosome organization. EMBO Rep..

[msu198-B13] Ceol CJ, Horvitz HR (2004). A new class of *C. elegans* synMuv genes implicates a Tip60/NuA4-like HAT complex as a negative regulator of Ras signaling. Dev Cell..

[msu198-B14] Chamberlin HM, Thomas JH (2000). The bromodomain protein LIN-49 and trithorax-related protein LIN-59 affect development and gene expression in *Caenorhabditis elegans*. Development.

[msu198-B15] Chen PJ, Cho S, Jin SW, Ellis RE (2001). Specification of germ cell fates by FOG-3 has been conserved during nematode evolution. Genetics.

[msu198-B16] Chen PJ, Ellis RE (2000). TRA-1A regulates transcription of *fog-3*, which controls germ cell fate in *C. elegans*. Development.

[msu198-B17] Cherry CM, Matunis EL (2010). Epigenetic regulation of stem cell maintenance in the *Drosophila* testis via the nucleosome-remodeling factor NURF. Cell Stem Cell.

[msu198-B18] Cho S, Jin SW, Cohen A, Ellis RE (2004). A phylogeny of *Caenorhabditis* reveals frequent loss of introns during nematode evolution. Genome Res..

[msu198-B19] Clapier CR, Cairns BR (2009). The biology of chromatin remodeling complexes. Annu Rev Biochem..

[msu198-B20] Clifford R, Lee MH, Nayak S, Ohmachi M, Giorgini F, Schedl T (2000). FOG-2, a novel F-box containing protein, associates with the GLD-1 RNA binding protein and directs male sex determination in the *C. elegans* hermaphrodite germline. Development.

[msu198-B21] Corona DF, Tamkun JW (2004). Multiple roles for ISWI in transcription, chromosome organization and DNA replication. Biochim Biophys Acta..

[msu198-B22] Cui M, Chen J, Myers TR, Hwang BJ, Sternberg PW, Greenwald I, Han M (2006). SynMuv genes redundantly inhibit lin-3/EGF expression to prevent inappropriate vulval induction in *C. elegans*. Dev Cell..

[msu198-B23] Cui M, Han M (2007). Roles of chromatin factors in *C. elegans* development.

[msu198-B24] Cutter AD, Yan W, Tsvetkov N, Sunil S, Felix MA (2010). Molecular population genetics and phenotypic sensitivity to ethanol for a globally diverse sample of the nematode *Caenorhabditis briggsae*. Mol Ecol..

[msu198-B25] Doniach T, Hodgkin J (1984). A sex-determining gene, *fem-1*, required for both male and hermaphrodite development in *Caenorhabditis elegans*. Dev Biol..

[msu198-B26] Duveau F, Félix MA (2012). Role of pleiotropy in the evolution of a cryptic developmental variation in *Caenorhabditis elegans*. PLoS Biol..

[msu198-B27] Ellis RE, Kimble J (1995). The *fog-3* gene and regulation of cell fate in the germ line of *Caenorhabditis elegans*. Genetics.

[msu198-B28] Fay DS, Yochem J (2007). The SynMuv genes of *Caenorhabditis elegans* in vulval development and beyond. Dev Biol..

[msu198-B29] Featherstone M (2002). Coactivators in transcription initiation: here are your orders. Curr Opin Genet Dev..

[msu198-B30] Felix MA, Braendle C, Cutter AD (2014). A streamlined system for species diagnosis in C*aenorhabditis* (nematoda: rhabditidae) with name designations for 15 distinct biological species. PLoS One.

[msu198-B31] Fire A, Xu S, Montgomery MK, Kostas SA, Driver SE, Mello CC (1998). Potent and specific genetic interference by double-stranded RNA in *Caenorhabditis elegans*. Nature.

[msu198-B32] Fodor A, Riddle DL, Nelson FK, Golden JW (1983). Comparison of a new wild-type *Caenorhabditis briggsae* with laboratory strains of *C. briggsae* and *C. elegans*. Nematologica.

[msu198-B33] Frohman MA (1994). On beyond classic RACE (rapid amplification of cDNA ends). PCR Methods Appl..

[msu198-B34] Gosney R, Liau WS, LaMunyon CW (2008). A novel function for the presenilin family member *spe-4:* inhibition of spermatid activation in *Caenorhabditis elegans*. BMC Dev Biol..

[msu198-B35] Grote P, Conradt B (2006). The PLZF-like protein TRA-4 cooperates with the Gli-like transcription factor TRA-1 to promote female development in *C. elegans*. Dev Cell..

[msu198-B36] Guo Y, Chen X, Ellis RE (2013). Evolutionary change within a bipotential switch shaped the sperm/oocyte decision in hermaphroditic nematodes. PLoS Genet..

[msu198-B37] Guo Y, Lang S, Ellis RE (2009). Independent recruitment of F box genes to regulate hermaphrodite development during nematode evolution. Curr Biol..

[msu198-B38] Haag ES (2005). The evolution of nematode sex determination: *C. elegans* as a reference point for comparative biology.

[msu198-B39] Hill RC, de Carvalho CE, Salogiannis J, Schlager B, Pilgrim D, Haag ES (2006). Genetic flexibility in the convergent evolution of hermaphroditism in *Caenorhabditis* nematodes. Dev Cell..

[msu198-B40] Hodgkin J (1986). Sex determination in the nematode *C. elegans:* analysis of *tra-3* suppressors and characterization of *fem* genes. Genetics.

[msu198-B41] Hodgkin JA, Brenner S (1977). Mutations causing transformation of sexual phenotype in the nematode *Caenorhabditis elegans*. Genetics.

[msu198-B42] Jin SW, Kimble J, Ellis RE (2001). Regulation of cell fate in *Caenorhabditis elegans* by a novel cytoplasmic polyadenylation element binding protein. Dev Biol..

[msu198-B43] Kelleher DF, de Carvalho CE, Doty AV, Layton M, Cheng AT, Mathies LD, Pilgrim D, Haag ES (2008). Comparative genetics of sex determination: masculinizing mutations in *Caenorhabditis briggsae*. Genetics.

[msu198-B44] Kimble J, Crittenden SL (2007). Control of germline stem cells, entry into meiosis, and the sperm/oocyte decision in *C. elegans*. Annu Rev Cell Dev Biol..

[msu198-B45] Kimble J, Edgar L, Hirsh D (1984). Specification of male development in *Caenorhabditis elegans:* the fem genes. Dev Biol..

[msu198-B46] Kiontke K, Gavin NP, Raynes Y, Roehrig C, Piano F, Fitch DH (2004). *Caenorhabditis* phylogeny predicts convergence of hermaphroditism and extensive intron loss. Proc Natl Acad Sci U S A..

[msu198-B47] Kiontke KC, Félix MA, Ailion M, Rockman MV, Braendle C, Penigault JB, Fitch DH (2011). A phylogeny and molecular barcodes for *Caenorhabditis*, with numerous new species from rotting fruits. BMC Evol Biol..

[msu198-B48] Kwon SY, Xiao H, Wu C, Badenhorst P (2009). Alternative splicing of NURF301 generates distinct NURF chromatin remodeling complexes with altered modified histone binding specificities. PLoS Genet..

[msu198-B49] Lamont LB, Kimble J (2007). Developmental expression of FOG-1/CPEB protein and its control in the *Caenorhabditis elegans* hermaphrodite germ line. Dev Dyn..

[msu198-B50] Lee MH, Kim KW, Morgan CT, Morgan DE, Kimble J (2011). Phosphorylation state of a Tob/BTG protein, FOG-3, regulates initiation and maintenance of the *Caenorhabditis elegans* sperm fate program. Proc Natl Acad Sci U S A..

[msu198-B51] Lo TW, Pickle CS, Lin S, Ralston EJ, Gurling M, Schartner CM, Bian Q, Doudna JA, Meyer BJ (2013). Using TALENs and CRISPR/Cas9 to engineer insertions and deletions. Genetics.

[msu198-B52] Montgomery MK, Xu S, Fire A (1998). RNA as a target of double-stranded RNA-mediated genetic interference in *Caenorhabditis elegans*. Proc Natl Acad Sci U S A..

[msu198-B53] Morgan CT, Noble D, Kimble J (2013). Mitosis-meiosis and sperm-oocyte fate decisions are separable regulatory events. Proc Natl Acad Sci U S A..

[msu198-B54] Nayak S, Goree J, Schedl T (2005). *fog-2* and the evolution of self-fertile hermaphroditism in *Caenorhabditis*. PLoS Biol..

[msu198-B55] Nelson GA, Lew KK, Ward S (1978). Intersex, a temperature-sensitive mutant of the nematode *Caenorhabditis elegans*. Dev Biol..

[msu198-B56] Ross JM, Zarkower D (2003). Polycomb group regulation of Hox gene expression in *C. elegans*. Dev Cell..

[msu198-B57] Schaner CE, Deshpande G, Schedl PD, Kelly WG (2003). A conserved chromatin architecture marks and maintains the restricted germ cell lineage in worms and flies. Dev Cell..

[msu198-B58] Schvarzstein M, Spence AM (2006). The *C. elegans* sex-determining GLI protein TRA-1A is regulated by sex-specific proteolysis. Dev Cell..

[msu198-B59] Snow JJ, Lee MH, Verheyden J, Kroll-Conner PL, Kimble J (2013). *C. elegans* FOG-3/Tob can either promote or inhibit germline proliferation, depending on gene dosage and genetic context. Oncogene.

[msu198-B60] Stein LD, Bao Z, Blasiar D, Blumenthal T, Brent MR, Chen N, Chinwalla A, Clarke L, Clee C, Coghlan A (2003). The genome sequence of *Caenorhabditis briggsae:* a platform for comparative genomics. PLoS Biol..

[msu198-B61] Thompson BE, Bernstein DS, Bachorik JL, Petcherski AG, Wickens M, Kimble J (2005). Dose-dependent control of proliferation and sperm specification by FOG-1/CPEB. Development.

[msu198-B62] Travers A (2000). Recognition of distorted DNA structures by HMG domains. Curr Opin Struct Biol..

[msu198-B63] Tsukiyama T, Daniel C, Tamkun J, Wu C (1995). ISWI, a member of the SWI2/SNF2 ATPase family, encodes the 140 kDa subunit of the nucleosome remodeling factor. Cell.

[msu198-B64] Tsukiyama T, Wu C (1995). Purification and properties of an ATP-dependent nucleosome remodeling factor. Cell.

[msu198-B65] Wei Q, Shen Y, Chen X, Shifman Y, Ellis RE (2014). Rapid creation of forward-genetics tools for *C. briggsae* using TALENs: lessons for nonmodel organisms. Mol Biol Evol..

[msu198-B66] Wood AJ, Lo TW, Zeitler B, Pickle CS, Ralston EJ, Lee AH, Amora R, Miller JC, Leung E, Meng X (2011). Targeted genome editing across species using ZFNs and TALENs. Science.

[msu198-B67] Woodruff GC, Eke O, Baird SE, Félix MA, Haag ES (2010). Insights into species divergence and the evolution of hermaphroditism from fertile interspecies hybrids of *Caenorhabditis* nematodes. Genetics.

[msu198-B68] Xi L, Fondufe-Mittendorf Y, Xia L, Flatow J, Widom J, Wang JP (2010). Predicting nucleosome positioning using a duration Hidden Markov Model. BMC Bioinformatics.

[msu198-B69] Xiao H, Sandaltzopoulos R, Wang HM, Hamiche A, Ranallo R, Lee KM, Fu D, Wu C (2001). Dual functions of largest NURF subunit NURF301 in nucleosome sliding and transcription factor interactions. Mol Cell..

[msu198-B70] Yen K, Vinayachandran V, Batta K, Koerber RT, Pugh BF (2012). Genome-wide nucleosome specificity and directionality of chromatin remodelers. Cell.

[msu198-B71] Zarkower D, Hodgkin J (1992). Molecular analysis of the *C. elegans* sex-determining gene *tra-1:* a gene encoding two zinc finger proteins. Cell.

[msu198-B72] Zhang H, Azevedo RB, Lints R, Doyle C, Teng Y, Haber D, Emmons SW (2003). Global regulation of Hox gene expression in *C. elegans* by a SAM domain protein. Dev Cell..

