# Supplementary material for: Dependence of the Sperm/Oocyte Decision on the Nucleosome Remodeling Factor Complex Was Acquired during Recent Caenorhabditis briggsae Evolution
Source: Mol Biol Evol. 2014 Jul 1;31(10):2573–85. doi: 10.1093/molbev/msu198 (PMC4166919; doi:10.1093/molbev/msu198)
Supplement: Supplementary Data [file supp_31_10_2573__index.html]

Dependence of the sperm/oocyte decision on the Nucleosome Remodeling Factor Complex was acquired during recent C. briggsae evolution — Dependence of the Sperm/Oocyte Decision on the Nucleosome Remodeling Factor Complex Was Acquired during Recent Caenorhabditis briggsae Evolution — Dependence of the Sperm/Oocyte Decision on the Nucleosome Remodeling Factor Complex Was Acquired during Recent Caenorhabditis briggsae Evolution — Supplementary Data 

# Dependence of the Sperm/Oocyte Decision on the Nucleosome Remodeling Factor Complex Was Acquired during Recent *Caenorhabditis briggsae* Evolution

## Supplementary Data

files

**Files in this Data Supplement:**

- Supplementary Data - pdf file
- Supplementary Data - pdf file
