# Supplementary material for: Dependence of the Sperm/Oocyte Decision on the Nucleosome Remodeling Factor Complex Was Acquired during Recent Caenorhabditis briggsae Evolution
Source: Mol Biol Evol. 2014 Jul 1;31(10):2573–85. doi: 10.1093/molbev/msu198 (PMC4166919; doi:10.1093/molbev/msu198)
Supplement: Supplementary Data [file supp_msu198_NURF_MBE_SUP.pdf]

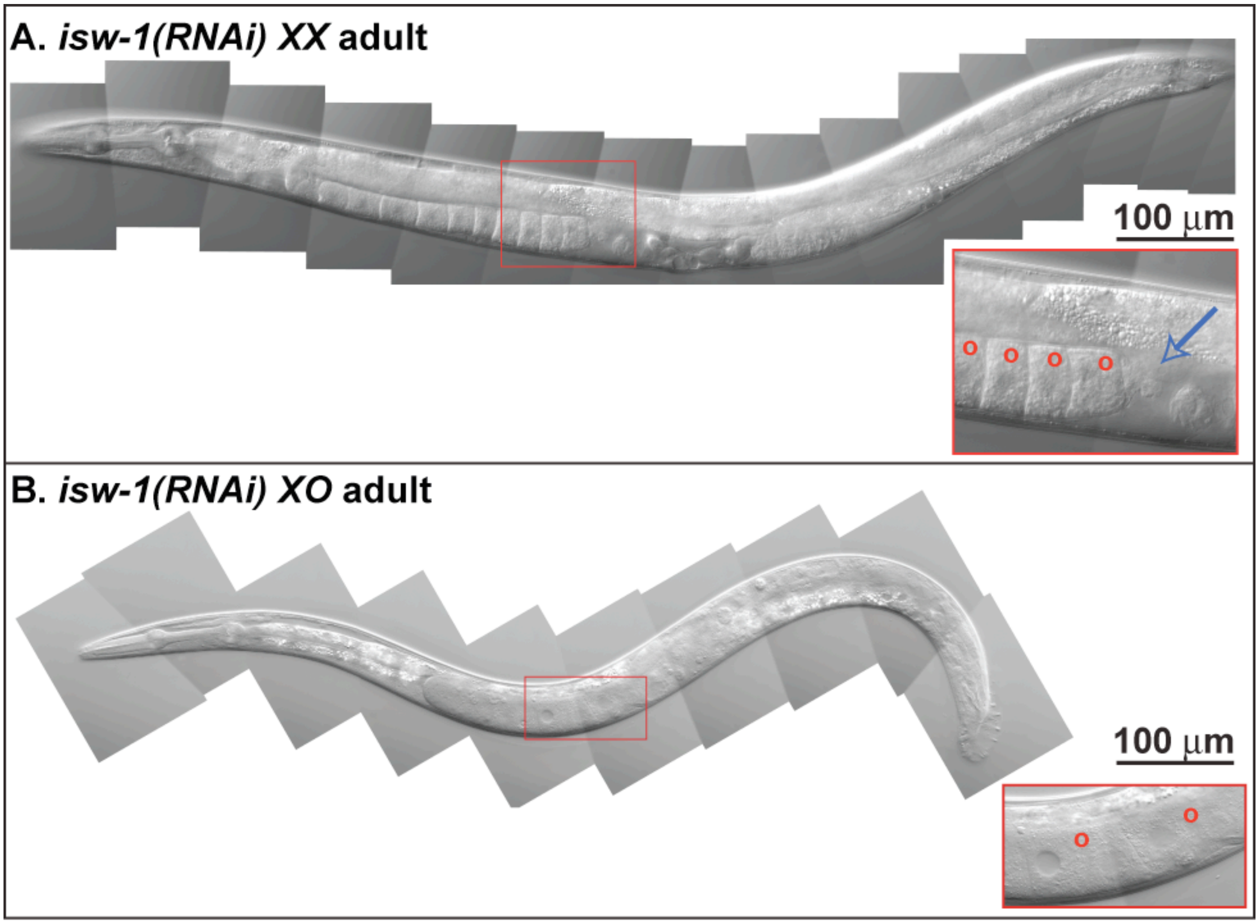

Figure S1. *C. briggsae* ISW-1 controls the sperm/oocyte decision.

A. *C. briggsae isw-1(RNAi) XX* Fog adult. B. *C. briggsae isw-1(RNAi) XO* Fog male. In both panels, anterior is left and ventral is down. The scale bars apply to the main panels; the size of each inset is shown by a box on the main panel. Finally, “o” indicates oocytes and a hollow blue arrow marks an empty spermatheca.

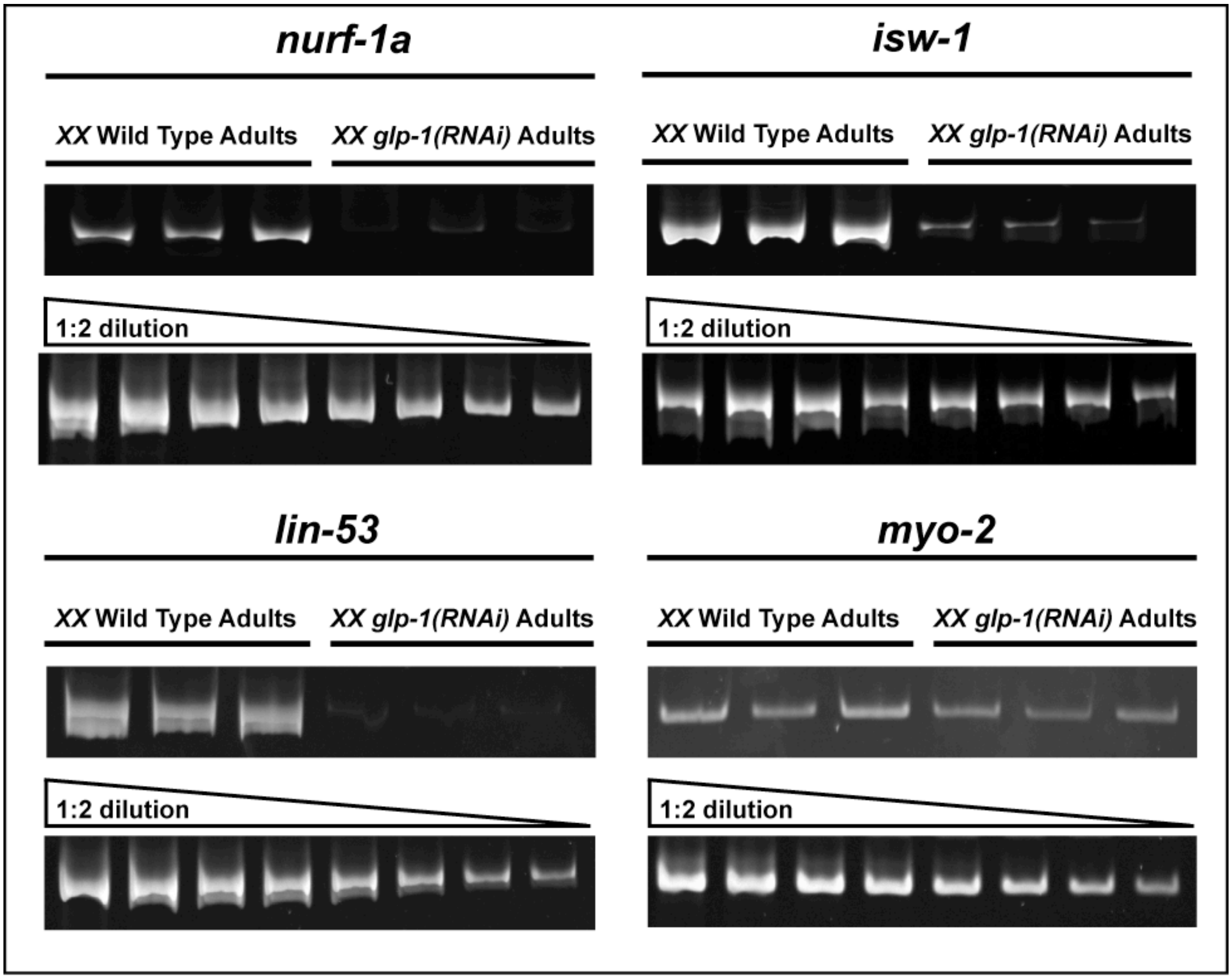

Figure S2. Components of the NURF complex are predominantly expressed in germ cells. Electrophoresis images of semi-quantitative RT-PCR experiments. Three biological replicates were assayed for each experiment (upper panels). Each lane contains equivalent amounts of cDNA isolated from a pool of five *XX* adults of the indicated genotype. The *glp-1(RNAi)* animals lacked germ cells. A 1:2 dilution series of wild-type cDNA template was tested for each target gene to show that the reactions were sensitive to changes in template concentration (lower panels). The endogenous control was *myo-2,* which is expressed exclusively in muscle.

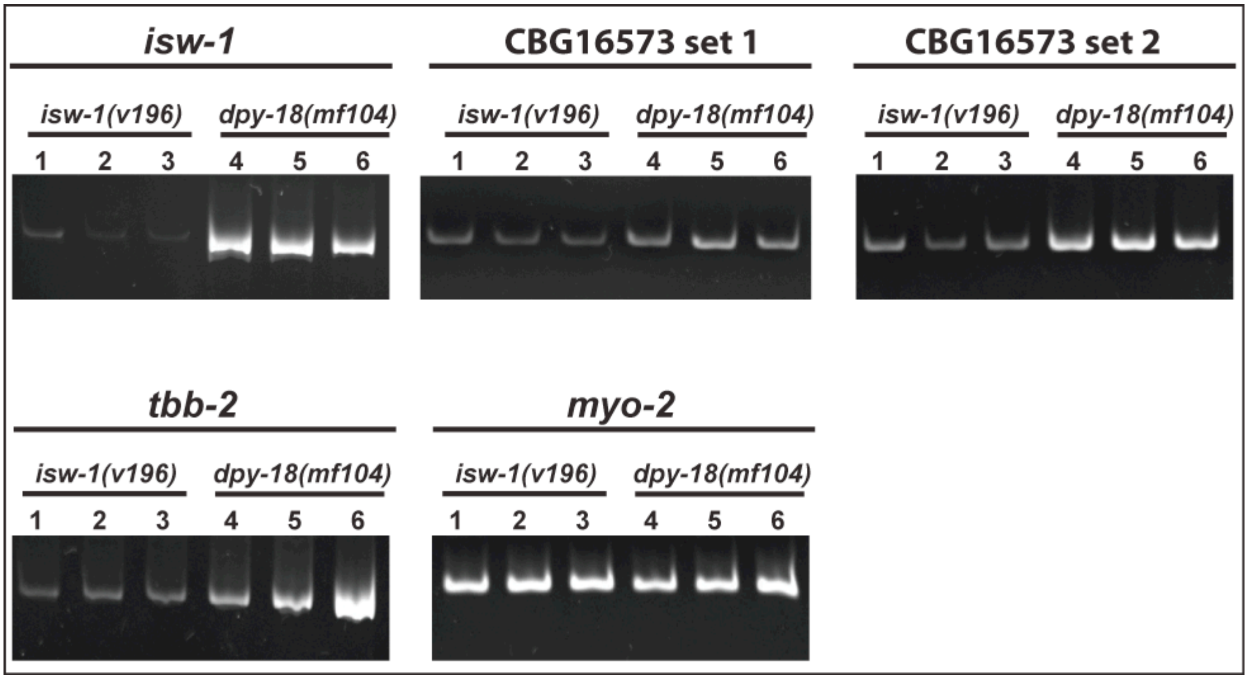

Figure S3. ***isw-1(v196)*** **is a null allele.**

Semi-quantitative RT-PCR analysis of samples from the homozygous *isw-1(v196)* progeny of *isw-1(v196) +/+ dpy-18* mothers, and from control *dpy-18* animals. Three independent biological replicates were used for each sample. The expression profiles of *isw-1* and the two nearby genes are shown in the top three panels, and that of two controls in the bottom panel. The *tbb-2* gene encodes a β-tubulin, and *myo-2* encodes a muscle-specific myosin heavy chain. The faint *isw-1* bands in *v196* are unspliced genomic DNA.

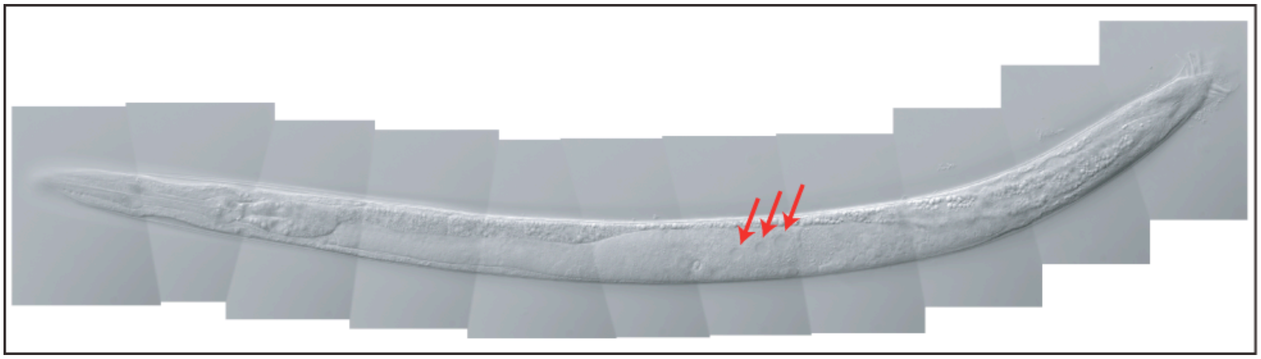

Figure S4. Lowering the dose of *cbr-isw-1* can cause oogenesis in males.

DIC photomiocrograph of an *isw-1(v196/+)* male, showing oocytes (red arrows). Anterior is to the left and ventral down.

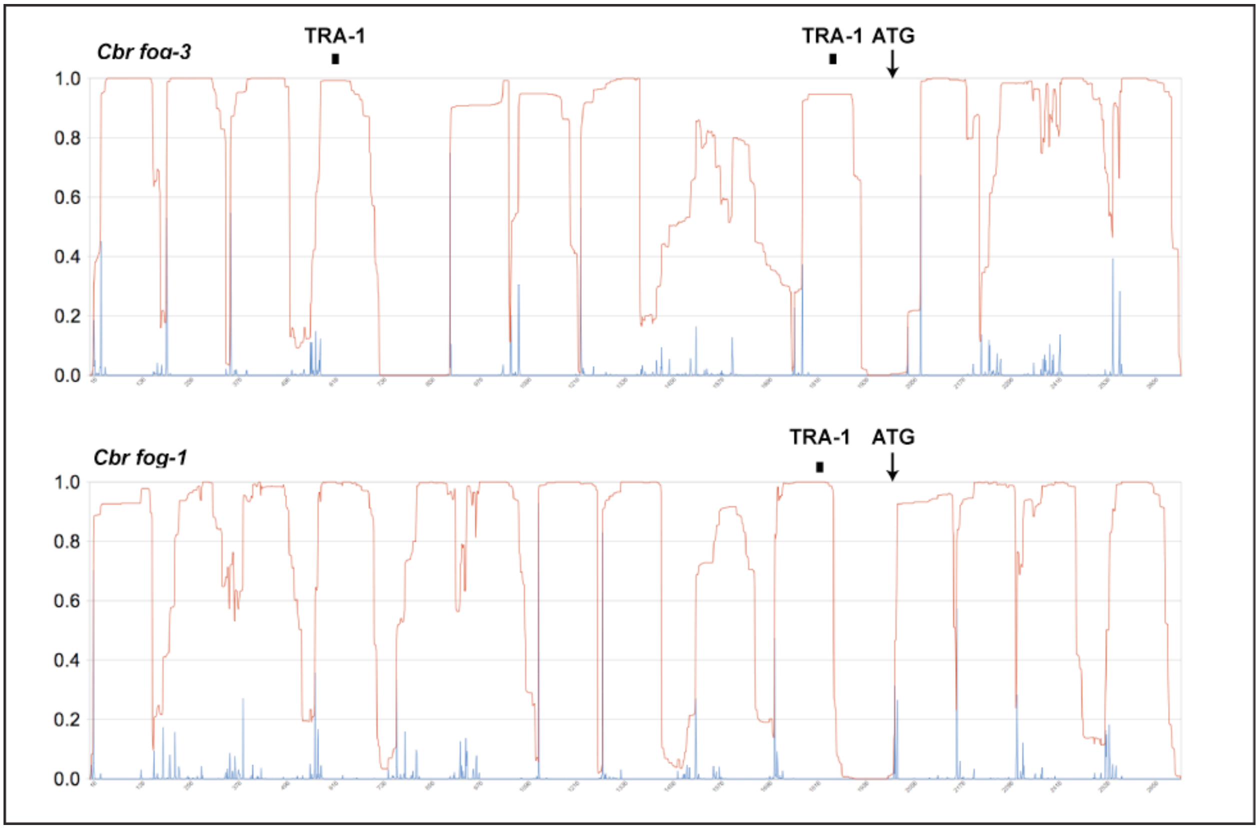

Figure S5. The TRA-1 binding sites in *Cbr-fog-1* and *Cbr-fog-3* are buried in nucleosomes. Graphs showing the beginnings of the *Cbr-fog-1* and *Cbr-fog-3* genes; each one includes 2000 nucleotides of genomic DNA upstream of the ATG. The Nucleosome Occupancy score is shown in red, and the probability that each base is the start of a nucleosome binding site is in blue (Xi et al., 2010). The predicted TRA-1 binding sites are marked by black line segments.

Table S1. No synthetic feminization between *C. elegans fog-1(q253*ts*)* and NURF RNAi

Genetic Background	RNAi Target	Fertile hermaphrodite	Fog	Sterile	Lethal	n
wild type	*Cel-nurf-1a*	98%	0%	0%	2%*	273
wild type	*Cel-isw-1*	32%	0%	67%†	1%*	384
Cel-fog-1(q253ts)	none	99.4%	0.6%	0%	0%	516
Cel-fog-1(q253ts)	*Cel-nurf-1a*	100%	0%	0%	0%	812
Cel-fog-1(q253ts)	*Cel-isw-1*	55%	0%	45%	0%	278

*- animals died as embryos

†- animals made sperm and abnormal oocytes

Table S2. RNAi targeting NURF-1 only causes a Fog phenotype in *C. briggsae XO* males

Species	Target	Oocytes only	Sperm & oocytes	Sperm only	Other	n
C. briggsae	*nurf-1a*	62%	9%	20%	9%*	44
C. briggsae	*nurf-1a*†	0%	50%	10%	40%*	20
C. briggsae	*isw-1*	0%	93%	0%	7%*	30
C. briggsae	*isw-1*‡	20%	4%	76%	0%	24
C. elegans	*nurf-1a*	0%	0%	98%	2%§	63
C. elegans	*isw-1*	0%	0%	100%	0%	28
C. sp. 9	*nurf-1a*	0%	0%	57%	42%‖	170
C. sp. 9	*nurf-1a*¶	0%	0%	46%	54%$^{\alpha}$	41
C. sp. 9	*isw-1*	0%	0%	40%	60%$^{\beta}$	82
C. sp. 9	*fog-3*	42%	0%	45%	13%$^{\gamma}$	38
C. remanei	*nurf-1a*	0%	0%	92%$^{\delta}$	8%$^{\varepsilon}$	66
C. remanei	*isw-1*	0%	0%	12%	88%$^{\zeta}$	58

* - Germ lines had undifferentiated germ cells, and some had vacuoles.

† - Injected with 1.0 mg/ml *C. sp. 9 nurf-1a* dsRNA.

‡ - Injected with 1.0 mg/ml *C. sp. 9 isw-1* dsRNA.

§ - Sperm leaked from the gonad.

‖ - 71 animals had undifferentiated germ cells; of these animals, 23 had abnormally small germ lines, 6 worms had ruptured gonads, and 3 worms had vacuoles in the germ line.

¶ - Injected with 0.5 mg/ml *C. sp. 9 nurf-1a* dsRNA.

α - 20 animals had small germ lines with undifferentiated germ cells, and 2 animals had disorganized germ lines with vacuoles.

β - These animals had small germ lines with undifferentiated germ cells.

γ - Undifferentiated germ cells.

δ - Out of these males, 10 had small germ lines with few sperm.

ε - These animals had small germ lines without sperm or spermatocytes.

ζ - 49 animals had small germ lines with undifferentiated germ cells, and 5 of these also had vacuoles. 2 animals had normal-sized germ lines with spermatocytes but no sperm.

Table S3. Primers used for RNA interference

Target	Sequence
Cbr-nurf-1a exon 2	F: TAATACGACTCACTATAGGGAGAAAATCCCGGAAGACCCGTCAAGAA<br>R: TAATACGACTCACTATAGGGAGATCCGTCACCGAATAATGCGTTTGC
Cbr-nurf-1a exon 7	F: TAATACGACTCACTATAGGGAGACTAGTCCGCAAGAAGCAAACTGAC<br>R: TAATACGACTCACTATAGGGAGACCATCTTCTTCACTTTCTGCTGTTCC
Cbr-nurf-1.2cef	F: TAATACGACTCACTATAGGGAGATCTGCGAACCTCTCCAAATCGGAA<br>R: TAATACGACTCACTATAGGGAGAGCAATACAGCGCTGGTTGTTCCTT
Cbr-isw-1	F: TAATACGACTCACTATAGGGAGATCTTGGAATCAACTTGGCAACCGC<br>R: TAATACGACTCACTATAGGGAGATGGCTCGATCCAGAAATGACCCAT
Cbr-lin-53	F: TAATACGACTCACTATAGGGAGAGAGCGTGCAATGGCTTCCAGAAAT<br>R: TAATACGACTCACTATAGGGAGATCTGGTCGTCTGAAGCGGAAAGAA
Cbr-rba-1	F: TAATACGACTCACTATAGGGAGAAATCTCTGGGATCTTCGTCACCCA<br>R: TAATACGACTCACTATAGGGAGAGGCGATTCGAGTTCCACGAGAAAT
Cbr-pyp-1	F: TAATACGACTCACTATAGGGAGATATCAAGCCGTTGAGCGTGGATCT<br>R: TAATACGACTCACTATAGGGAGAATCAGTGCCAATGTTCCGAGGACT
Cel-nurf-1a	F: TAATACGACTCACTATAGGGAGACAGGATATTCCGATTCCAACGGCT<br>R: TAATACGACTCACTATAGGGAGAAGTAGTGCGTCTGCTCTTCATCGT
Cel-isw-1	F: TAATACGACTCACTATAGGGAGATGGAATCAACTTGGCTACCGCTGA<br>R: TAATACGACTCACTATAGGGAGAAGAAATGACCCATTCCGTCGGCTT
Cre-nurf-1a	F: TAATACGACTCACTATAGGGAGATCCCAAAGCTAGACCTTCCAGAGA<br>R: TAATACGACTCACTATAGGGAGAGTATCAGCAAACGGATACGCCTCA
C. sp.9 nurf-1a exon 2	F: TAATACGACTCACTATAGGGAGAAATCCCGGAAGAGCCGTCAAGAA<br>R: TAATACGACTCACTATAGGGAGAACCGAATAATGCGTCTGCTCCTCA
C. sp.9 fog-3	Fa: TAATACGACTCACTATAGGGAGAAGTTCCGAGAAGAGCAACCGGAAT<br>Ra: TAATACGACTCACTATAGGGAGAGCGAATGTGGGAAAGGTTCGAGTT<br>Fb: TAATACGACTCACTATAGGGAGAAGTTCCGAGAAGAGCAACCGGAAT<br>Rb: TAATACGACTCACTATAGGGAGATGTTGTTGACCAGTTCGAGCACAG

Table S4A. Primers used for semi-quantitative RT-PCR

Target	Sequence
Cbr-nurf-1a	F: AAATCCCGGAAGACCCGTCAAGAA<br>R: TCCGTCACCGAATAATGCGTTTGC
Cbr-isw-1	F:TAATACGACTCACTATAGGGAGATCTTGGAATCAACTTGGCAACCGC<br>R:TAATACGACTCACTATAGGGAGATGGCTCGATCCAGAAATGACCCAT
Cbr-lin-53	F:TAATACGACTCACTATAGGGAGAGAGCGTGCAATGGCTTCCAGAAAT<br>R:TAATACGACTCACTATAGGGAGATCTGGTCGTCTGAAGCGGAAAGAA
Cbr-myo-2	F: AAGGTGCTGCCAGAGTAACATTCG<br>R: GAAACTTGCGAGCACCGGTTTCAA

Table S4. Primers used for real-time quantitative RT-PCR

Target	Sequence
Cbr-fog-1	F: GCTTCCGAGTTTTGAGTGTG<br>R: GCTGGTAGGAGAATATGTTGG
Cbr-fog-3	F: CCTAGAAATGGTCAAATGGAGAG<br>R: TTCTGGAATTGGCTGATAGTG
Cbr-spe-4	F: AATTGGACAACCGGGAATC<br>R: GCCCAAACATCCATAGACAAC
Cbr-rpb-1	F: CGACAACCCACTCTCCATAA<br>R: GCCAATCGATGAAGATGTCAC
Cbr-nurf-1a	F: CCGTTTCCAGACATCCTAGT<br>R: GAGAAGTTGGTACAGTTGAGG
Cbr-isw-1	F: ACAGGCAGAACAAATGGG<br>R: GCTGGAACGTCTTTTGGTC
