# Supplementary material for: Dependence of the Sperm/Oocyte Decision on the Nucleosome Remodeling Factor Complex Was Acquired during Recent Caenorhabditis briggsae Evolution
Source: Mol Biol Evol. 2014 Jul 1;31(10):2573–85. doi: 10.1093/molbev/msu198 (PMC4166919; doi:10.1093/molbev/msu198)
Supplement: Supplementary Data [file supp_msu198_MBE_Response_to_Reviewers.pdf]

We thank both reviewers for detailed and thoughtful comments. We made changes to address all of their concerns, and detail these revisions below.

Reviewer 1

(1) Page 6, 1st paragraph: a more complete description of the *C. briggsae* NURF and ISWI genes should be given in this paragraph. In particular, it should be stated that the ortholog of *cel-nurf-1* appears to be separated into two distinct genes in *C. briggsae,* CBG11092, which codes for the *cbr-nurf-1a* transcript and CBG11091+CBG11090, which codes for the *cbr-nurf-1cef* transcripts. These two genes should be given distinct names as there is no evidence for a single transcript that contains sequences from both of these regions. Moreover, the presence of SL1 at the beginning of *cbr-nurf-1c* suggests that these transcripts do not arise from a single trans-spliced operon. Finally, analyses in this manuscript indicate that these genes have distinct functions. [It also would be helpful if the manuscript referred to the gene predictions corresponding to the *cbr-nurf* and *cbr-isw-1* genes.]

Some colleagues favored naming *Cbr-nurf-1* as a single gene (as we did in this manuscript) and others as two genes (as we did at a meeting last year, and as this reviewer recommends). Thus, we consulted Paul Sternberg, head of WormBase, and Tim Schedl, who is in charge of nomenclature for WormBase, and they discussed this issue with their staff. They recommended using the single name *Cbr-nurf-1* for the entire locus, because all of the *C. briggsae nurf-1* transcripts are orthologous to *C. elegans nurf-1* transcripts. Hence, the name *Cbr-nurf-1* most clearly reflects the evolutionary relationships.

However, they also recommended defining two genes within this complex locus, named *nurf-1A* (which encodes the *nurf-1a* transcript) and *nurf-1B* (which encodes the other transcripts). Tim Schedl went over Figure 1 and approved the way we presented the nomenclature. Since the A and B designations reflect the transcriptional organization, Tim Schedl recommended using them only when it was important to compare one half of the locus with the other.

Thus, we rewrote the passage starting at the bottom of page 6 to read: "When we used RT-PCR to characterize the *C. briggsae* messages, we detected four *nurf-1* transcripts (Fig. 2D), which correspond to prominent *nurf-1* transcripts identified in *C. elegans* (Andersen et al., 2006). One message is produced from the left half of the *nurf-1* locus, which we name *nurf-1A*. The remaining transcripts are produced from the right half of the locus, which we call *nurf-1B*. Neither *C. briggsae* nor *C. elegans* makes a product like full-length *Drosophila* NURF301, which would span the entire region (Andersen et al., 2006; Xiao et al., 2001), so the range of NURF-1 isoforms differs from that seen in fruit flies."

We also added the following text to the Methods section **Determination of gene structures**: "Because of the duplication and specialization of two exons, the genomic sequences that encode the *C. briggsae nurf-1a* transcript do not overlap those that encode the remaining *nurf-1* transcripts. We are naming this entire complex locus *"nurf-1"* to be consistent with the literature from other species. The portion that encodes the *nurf-1a* transcript is officially the *nurf-1A* gene. The portion that encodes the remaining *nurf-1* transcripts is *nurf-1B*."

We also submitted our data to wormbase, and are working with them to update the gene models presented for *nurf-1* so that they will reflect our results.

(2) Figure 1 C and E: the % Fog and % Lethality data in figure 1 is redundant with Table 1. There also is a slight inconsistency between Figure 1 and Table 1. Figure 1C indicates that %Fog values for all isw-1 alleles were obtained at 15 ˚C. Table 1 states that only the data for isw-1(v183cs) was obtained at 15 ˚C. These data should be deleted from Figure 1.

We removed the percentages from Figure 1, and simply listed the types of phenotypes that were observed, with a reference in the legend to see Table 1. We checked Table 1 and it is accurate.

(3) Page 8, paragraph on *Cbr-nurf-1a* results: it should be mentioned that *Cbr-nurf-1a* is the only transcript that encodes a protein that contains an HMGA domain. As the model presented by the authors for *Cbr-nurf-1a* function involves chromatin remodeling, the presence of this domain should be noted in the results and discussed when the model is presented.

Thank you for pointing out the importance of mentioning this feature of NURF-1A here.

We added this sentence to the Results: "NURF-1A is also the only isoform to contain an HMGA domain, which is known to bind DNA (Travers, 2000)."

We modified this passage in the Discussion: "Two types of models could explain this change. In the first, the *C. briggsae* NURF complex was recruited to regulate the sperm/oocyte decision; NURF-1A might be critical for this function because it has an HMGA domain, which can bind DNA."

(4) Page 8: the sentence "As in *C. elegans, Cbr-isw-1* is trans-spliced to SL1 and produces a single transcript, and RNAi against the remaining NURF components was lethal" does not make sense. It appears that something was inadvertently deleted to fuse two sentences together and eliminate much of the *Cbr-isw-1* RNAi results.

The corrected paragraph now reads: "As in *C. elegans, Cbr-isw-1* is trans-spliced to SL1, and produces a single transcript. When we used RT-PCR to study *nurf-1a, isw-1* and *lin-53* transcript levels, we found that each was predominantly expressed in germ cells, as expected from the phenotypes we observed (Fig. S2)."

We moved the ectopic sentence to the section **The *C. briggsae* NURF complex is required to initiate spermatogenesis in both sexes**, where it belongs: "As in *C. elegans,* RNAi against the remaining NURF components was lethal (Table 2)."

Reviewer: 2

---

(1) Alternative splicing of NURF / nurf-1 in nematodes: In wormbase there are seven transcripts of nurf-1 for *C. elegans*. I am uncertain why the authors comment / spend time trying to identify a "full-length" nurf-1 splice variant as compared to *Drosophila*. If the authors think this is important (the absence of the "full-length" transcript found in flies) there needs to be a better

justification. To me, this seems to be a bit of a non sequitor and I think it just confuses things. From the seven splice variants annotated in *C. elegans*, it is obvious that alternative splice variants are produced in nematodes as they are in flies.

We rewrote this section to clarify our point, as described above in the response to reviewer #1.

(2) Same section: I think the section title "A single NURF-1 product regulates developmental decisions in nematodes" is too broad – I understand what you mean, but I would prefer more precise language – nurf-1 regulates a germ cell fate change – this is a very specific developmental decision, rather than having pleiotropic affects in many cell types (I understand that there are also phenotypes causing a Pvl, but the authors do not follow these up).

We changed the title of this section to "**A single NURF-1 product regulates the sperm/oocyte decision in *C. briggsae*"**

(3) Timing – It would be nice to add in based on the literature the timing of these evolutionary changes. There are several times in the text where the authors refer to these changes as rapid, but it would be good to have a frame of reference. These could be added into the phylogeny in figure 4B and mentioned in the text where appropriate if these divergence times have been estimated.

Changing the figure would be hard, since some divergence times are not known. However, we added a reference in the Discussion to address this point. The paragraph now reads: "*C. nigoni* is so closely related to *C. briggsae* that they can mate and produce viable offspring (Woodruff et al., 2010), and these species appear to have diverged on the order of 10^7 generations ago (Cutter et al., 2010). However, knocking down NURF activity in *C. nigoni* did not cause males to make oocytes, and similar experiments with the more distant relative *C. remanei* had the same result."

(4) Figures:

(a) Figure 2: Reference scale bar in legend, provide scale bar for inset micrographs

Done.

(b) Figure 3: Adjust bar graphs vs. background so they are consistent (e.g., in Fig. 3B the top graph and bottom graph bars are different – you can see the background lines in the top graph, and not in the bottom).

Done.

(c) What statistical tests were performed to determine p-values reported in the figures and manuscript? This should be included in the text and figure legends along with the p-values as well as in the methods.

We added this to legend 3B: "P values were determined with a Student's t-Test, 1-tailed, with unequal variance."

We added this to legend 5C: “Error bars represent 95% confidence intervals for a proportion.”

We added this to the Methods: “**Statistical analyses**. To determine the significance of the difference between two proportions, we used www.vassarstats.net/propdiff_ind.html to calculate the z-ratio and p values.”

(d) Figure 5: Please fix typo : “Since NURF is known to remodel chromatin, the simplest possibility is opens up the fog-3 promoter, so that TRA-1 binding sites (shown in red) are accessible.”

We corrected the text to read: “Since NURF is known to remodel chromatin, the simplest possibility is that it opens up the *fog-3* promoter”
